# Integrated serum metabolomics reveals severity-associated exploratory metabolic signatures in burn patients

**DOI:** 10.3389/fphys.2026.1846440

**Published:** 2026-06-17

**Authors:** Zhenyu Cheng, Kang Li, Shaoyun Ran, Liming Zhang, Xiaoliang Li, Dawei Han, Shuai Wang, Leifeng He, Mengdan Xu, Meng Li, Haiying Huang, Haiping Di

**Affiliations:** 1 Zhengzhou First People’s Hospital, Zhengzhou, China; 2 Henan University of Chinese Medicine, Zhengzhou, China

**Keywords:** burn injury, exploratory candidate biomarkers, metabolomics, ROC analysis, UPLC-MS/MS

## Abstract

**Objective:**

To systematically characterize the serum metabolome of burn patients using ultra-performance liquid chromatography-tandem mass spectrometry (UPLC-MS/MS). Patients were stratified by burn severity based on total body surface area (TBSA; ≥30% vs. <30%). Exploratory metabolic signatures and candidate biomarkers associated with burn severity were investigated.

**Methods:**

Peripheral serum samples were collected from patients with TBSA ≥30% (n=33), TBSA <30% (n=28), and healthy controls (n=18). Serum metabolomic profiling was performed by UPLC-MS/MS. Principal component analysis (PCA) and orthogonal partial least squares-discriminant analysis (OPLS-DA) were applied to identify differential metabolites. Pathway enrichment analysis was conducted using the Kyoto Encyclopedia of Genes and Genomes (KEGG) database via MetaboAnalyst. Receiver operating characteristic curve (ROC) analysis was performed to preliminarily evaluate the discriminatory performance of exploratory candidate biomarkers.

**Results:**

Distinct metabolic dysregulation was observed between burn severity groups. Compared with the healthy control group, twenty-three significantly altered metabolites were identified in patients with TBSA ≥30%, while thirty-one metabolites were altered in patients with TBSA <30%. Severe burns (TBSA ≥30%) predominantly disrupted amino acid and glycerophospholipid metabolism, whereas moderate burns (<30%) mainly involved lipid-related pathways. Patients with TBSA ≥30% exhibited perturbations across a broader range of metabolic pathways. ROC analysis identified nine exploratory candidate biomarkers (area under the curve [AUC] >0.8) in patients with TBSA ≥30% and eight in patients with TBSA <30%, suggesting preliminary discriminatory potential within the current cohort for distinguishing metabolic alterations associated with different burn severities.

**Conclusion:**

Burn severity may be associated with distinct serum metabolomic signatures. Key pathways and exploratory candidate biomarkers associated with burn severity-related metabolic alterations were identified. These findings provide a metabolomics-based reference framework for future studies investigating burn severity-associated metabolic alterations and may support future mechanistic and translational research in burn care.

## 1 Introduction

Burn injuries are among the most severe forms of trauma, characterized by full-thickness destruction of the skin, subcutaneous tissue, and sometimes deeper structures (Jeschke et al., 2020). They cause not only localized tissue damage but also intense systemic stress responses, including hypermetabolism, oxidative stress, immunosuppression, and multiple organ dysfunction (Parihar et al., 2008; Begum et al., 2022; Willis et al., 2022; Legrand et al., 2024). Total body surface area (TBSA) burned is widely recognized as a key prognostic indicator in clinical practice. When TBSA reaches ≥30%, patients are more likely to develop systemic inflammatory response syndrome (SIRS), infection, and multiple organ failure, with a markedly increased risk of mortality (Loss et al., 2000; Brusselaers et al., 2010; Stanojcic et al., 2018). Although burns may induce a variety of metabolic alterations, systematic studies comparing metabolic changes across different burn severities remain limited. Therefore, further exploratory investigations of metabolic patterns across different burn severities may improve understanding of burn-related pathophysiological processes and potentially inform future therapeutic strategies.

In recent years, metabolomics, as a vital component of systems biology, has become a powerful tool for exploring trauma-related metabolic alterations. In burn research, serum metabolomics has been widely used to explore biomarkers associated with burn severity, complications, and prognosis. These findings provide preliminary insights that may support future personalized diagnostic and therapeutic strategies (Loss et al., 2000; Stanojcic et al., 2018). Su et al (Su et al., 2024) applied metabolomics to examine the dynamic changes in metabolic profiles of severely burned patients. Their results quantified the temporal evolution of metabolic processes and revealed the long-term remodeling of metabolic networks after burns. Malachowska et al (Malachowska et al., 2023) analyzed hepatic metabolic responses in aged and young mice after burns. Their work showed marked age-related differences in metabolic adaptations. Collectively, these studies highlight the critical role of metabolomics in uncovering the metabolic characteristics and mechanisms underlying burn injury.

Ultra-performance liquid chromatography-tandem mass spectrometry (UPLC-MS/MS) offers high sensitivity, high resolution, and excellent quantitative reproducibility, making it one of the mainstream methods in serum metabolomics research. Moreover, UPLC-MS/MS can accurately capture dynamic changes in complex metabolic networks following burns, providing essential data for mechanistic studies and early diagnosis (Wang et al., 2023, 2023; Liu et al., 2025; Gu et al., 2024). In this study, serum metabolomics analysis was conducted on burn patients stratified by TBSA ≥30% and <30%, alongside a healthy control group. The aim was to characterize burn severity-associated metabolic alterations and to provide a metabolomics-based reference framework for future mechanistic and translational studies in burn patients.

## 2 Materials and methods

### 2.1 Experimental reagents and instruments

Methanol and acetonitrile, chromatographic grade (Thermo Fisher Scientific, USA); formic acid, mass spectrometry grade (Merck, Germany).

UltiMate 3000 UHPLC system coupled to an Orbitrap Exploris 240 high-resolution mass spectrometer (Thermo Fisher Scientific, USA); Multifuge X1R high-speed refrigerated centrifuge (Thermo Fisher Scientific, USA); SPD2030–230 centrifugal concentrator and dryer (Thermo Fisher Scientific, USA).

### 2.2 Patient recruitment and clinical data collection

Burn Patients: Peripheral blood samples were collected from male and female burn patients aged 18–60 years: 33 patients with TBSA ≥30% and 28 patients with TBSA <30%, admitted to Zhengzhou First People’s Hospital Burn Center between July 2024 and May 2025. At the Zhengzhou First People’s Hospital Burn Center, patients with ≥30% TBSA consistently exhibit more pronounced systemic metabolic disturbances and complex complications than those with <30% TBSA. Therefore, ≥30% TBSA was adopted as a clinically meaningful threshold for exploring metabolic dysregulation associated with severe burns in this cohort. Additionally, this cutoff allowed for a relatively balanced distribution of patients between subgroups for metabolomic comparisons. Informed consent was obtained from all participants or their legal representatives. Exclusion criteria were: 1) Age <18 years; 2) Specific burn types, including chemical or electrical burns; 3) Severe pre-existing complications, such as cardiovascular, hepatic, or renal diseases; 4) Endocrine disorders, including diabetes; 5) Neoplastic diseases; 6) Obesity (BMI ≥30); 7) Psychiatric disorders, cognitive impairment, or participation in other clinical trials.

Healthy Adult Volunteers: Eighteen healthy male and female adults aged 20–52 years were recruited from individuals undergoing routine physical examinations at Zhengzhou First People’s Hospital. Inclusion criteria were: 1) Overall good health; 2) No history of long-term medication use; 3) No major illnesses or recent infections; 4) No history of substance abuse or alcoholism.

### 2.3 Patient serum collection and sample preparation

Peripheral blood samples were collected within 24 h of patient admission. Blood samples were centrifuged at 3,500 rpm for 10 min. The supernatant was aliquoted and stored at −80°C. Peripheral blood samples from healthy volunteers were processed using the same procedure.

For metabolomic analysis, serum samples were thawed at 4°C. A 200 μL aliquot of serum supernatant was transferred to a new tube. Methanol (four volumes relative to serum volume, v/v) was added, followed by vortex mixing and sonication on ice for 10 min. Samples were then incubated at −20°C for 1 h to ensure complete protein precipitation. Subsequently, samples were centrifuged at 14,000 rpm and 4°C for 15 min. The supernatant was transferred to a centrifugal concentrator and evaporated to dryness. The residue was resuspended in 200 μL of methanol, vortexed, and centrifuged twice at 14,000 rpm and 4°C. The resulting supernatant was used for UPLC-MS/MS analysis. Quality control (QC) samples were prepared by pooling equal volumes of extracted supernatants from all samples. QC samples were injected at the beginning of the analytical sequence for system conditioning and subsequently analyzed after every 10 study samples throughout the run to monitor instrument stability and analytical reproducibility. Blank samples consisting of pure methanol extraction solvent were injected after every 10 sample injections to evaluate potential carryover and background contamination. A pooled QC-based strategy was applied to assess chromatographic reproducibility, retention time stability, and signal consistency during data acquisition. Prior to formal sample analysis, system suitability was evaluated using repeated QC injections, and only runs showing stable retention time, peak intensity, and mass accuracy were accepted for downstream analysis.

### 2.4 UPLC-MS/MS experiment

Chromatographic Conditions: Separation was performed using a Hypersil GOLD™ VANQUISH column (100 mm×2.1 mm, 1.9 μm). The mobile phase consisted of 0.1% formic acid in water (A) and acetonitrile (B). The flow rate was 0.2 mL/min, the injection volume was 2 μL, and the column temperature was 40°C. The gradient elution program was as follows: 0–1 min, 3% B; 1–8 min, 3–50% B; 8–24 min, 50–95% B; 24–26 min, 95% B; 26–27 min, 95–3% B; 27–30 min, 3% B.

Mass Spectrometry Conditions: Electrospray ionization (ESI) was applied in both positive ionization (ESI+) and negative ionization (ESI−) modes. Spray voltages were 3.5 kV (ESI+) and 3.0 kV (ESI−). The optimized ion source parameters were as follows: ion transfer tube temperature 350°C, vaporizer temperature 300°C, sheath gas 35 arbitrary units, auxiliary gas 10 arbitrary units, full scan resolution 60,000, carrier gas N_2_, and *m/z* range 100–1200. MS/MS data were acquired using data-dependent MS^2^ (ddMS^2^) mode. Fragmentation was performed using higher-energy collisional dissociation (HCD) with stepped normalized collision energies of 20, 40, and 60 eV. The precursor ion isolation window was set to 2 Da. The automatic gain control (AGC) target was set to standard mode, and the maximum injection time was set to auto mode. Dynamic exclusion was enabled with an exclusion duration of 60 s. Precursor ions were automatically selected based on signal intensity for MS/MS acquisition. LC-MS data were acquired in both ESI+ and ESI− modes using polarity switching within a single analytical run.

### 2.5 Metabolite annotation and identification confidence

Metabolite annotation was performed based on accurate mass measurement, isotope distribution, adduct pattern recognition, chromatographic retention behavior, and MS/MS fragmentation matching using the Human Metabolome Database (HMDB, https://hmdb.ca/), the Kyoto Encyclopedia of Genes and Genomes (KEGG, https://www.genome.jp/kegg/), and in-house spectral databases. Peak extraction, alignment, and normalization were performed using Compound Discoverer 3.3 software (Thermo Fisher Scientific).

The precursor ion mass tolerance was set to within 10 ppm, and fragment ion matching tolerance was set within 20 ppm. Common adduct forms, including [M+H]^+^, [M+Na]^+^, [M-H]^−^, and [M+Cl]^−^, were considered during metabolite annotation. Isotope peak patterns and retention time consistency across QC samples were also evaluated to improve annotation reliability.

MS/MS spectral matching was performed by comparing fragmentation patterns with public and commercial spectral databases. Authentic chemical reference standards were not used in the present study. According to the Metabolomics Standards Initiative (MSI) criteria, all reported metabolites were classified as MSI level 2, representing putatively annotated compounds based on MS/MS spectral similarity and database matching.

### 2.6 Multivariate statistical analysis

LC-MS data from serum samples of healthy adults and burn patients were acquired using UPLC-MS/MS in both ESI+ and ESI− modes. Data from ESI+ and ESI− modes were separated into two independent datasets for subsequent analysis. Raw UPLC-MS/MS data were imported into Compound Discoverer 3.3 software for standardization and normalization, including peak extraction, retention time (*t*_R_) correction, and normalization, to construct a data matrix. Peak alignment and metabolite annotation were performed using the integrated workflow of Compound Discoverer 3.3. Adduct ions, isotope peaks, and background contaminants annotated from blank samples were filtered during data processing to reduce redundant and non-biological signals. Features with poor analytical stability were excluded if they showed a relative standard deviation (RSD) >30% in pooled QC samples or were detected in less than 80% of QC injections. Missing values were imputed using half of the minimum detected value for the corresponding metabolite. Signal drift correction and inter-batch normalization were performed based on pooled QC samples to minimize analytical variation during long-sequence acquisition. Data were subsequently log-transformed and Pareto-scaled prior to multivariate statistical analysis. The resulting matrix was imported into SIMCA-P (version 14.1) software for principal component analysis (PCA) and orthogonal partial least squares-discriminant analysis (OPLS-DA). Differential metabolites were initially screened based on variable importance in projection (VIP) scores >1, Student’s *t*-test (*P* < 0.05), and fold change (FC) >1.2 or <0.83. To control for multiple- comparison bias, false discovery rate (FDR) correction was subsequently performed using the Benjamini-Hochberg method, and adjusted *P* values were calculated for all differential metabolites. Metabolites with adjusted *P* < 0.05 were considered statistically significant after multiple- comparison correction. In the primary analysis, metabolites were identified separately by comparing each burn group (TBSA ≥30% and TBSA <30%) with the healthy control group. To further evaluate severity-associated metabolic alterations, an additional direct comparison between the TBSA ≥30% and TBSA <30% burn groups was performed during downstream interpretation of exploratory severity-associated metabolic patterns.

### 2.7 Screening of exploratory candidate biomarkers and metabolic pathway analysis

Differential metabolites were annotated using the HMDB and the KEGG databases, to identify exploratory candidate biomarkers associated with burn severity. MetaboAnalyst (https://www.metaboanalyst.ca/) was employed for metabolic pathway analysis of the annotated exploratory candidate biomarkers, enabling the identification of key metabolic pathways. The biological significance of exploratory candidate biomarkers involved in these pathways was further assessed. MetaboAnalyst is a web-based metabolomics visualization and analysis tool designed to detect significantly altered metabolic pathways.

### 2.8 Receiver operating characteristic curve (ROC) analysis of exploratory candidate biomarkers

To preliminarily evaluate the discriminatory performance of exploratory candidate biomarkers identified in the current cohort, ROC analysis was conducted using GraphPad Prism 8.0 software. Exploratory candidate biomarkers with potential discriminatory value were identified based on an area under the curve (AUC) >0.8. The ROC curve illustrates the sensitivity and specificity of individual exploratory candidate biomarkers in distinguishing burn severity. Because of the relatively limited sample size and the exploratory nature of this untargeted metabolomics study, ROC analyses were performed only within the discovery cohort and no internal cross-validation, bootstrapping procedure, or independent external validation cohort was applied. Therefore, this analysis was intended to provide preliminary assessment of exploratory candidate biomarkers discriminatory performance rather than formal biomarker validation.

### 2.9 Statistical analyses

Patient demographic data were examined using GraphPad Prism software version 8.0. Differences in gender were determined by using Fisher’s exact test. Univariate analysis was performed using Student’s *t*-test or Mann-Whitney U test, as appropriate. To account for multiple comparisons in metabolomics analyses, FDR correction was applied using the Benjamini-Hochberg procedure, and adjusted *P* values were calculated for differential metabolites. An adjusted *P* < 0.05 was considered statistically significant after multiple-comparison correction. ROC analyses were performed to evaluate the discriminatory performance of exploratory candidate biomarkers within the current cohort, while metabolic pathway analyses were conducted to identify functional pathways associated with exploratory candidate biomarkers. A *P* < 0.05 was considered the threshold for statistical differences.

## 3 Results

### 3.1 Study population and characteristics

This study included 79 participants: 18 healthy adult volunteers, 33 burn patients with ≥30% TBSA, and 28 burn patients with <30% TBSA. The demographic and clinical characteristics of all participants are summarized in **Table 1**. The healthy control group consisted of 9 males and 9 females, with a median age of 35.5 years (range, 20–52 years). The ≥30% TBSA burn group had a median age of 50 years (range, 18–60 years) and a median TBSA of 50% (range, 32–97%). The <30% TBSA burn group had a median age of 39 years (range, 18–60 years) and a median TBSA of 15% (range, 6–28%). All burn injuries were thermal, caused by hot liquids or flames. Peripheral blood samples were collected during the acute admission phase using a consistent sampling protocol across all burn patients.

**Table 1:** Demographic and clinical characteristics of study participants.

Items	Controls	≥30% TBSA	<30% TBSA	*P*
Sex (Male/Female)	9/9	17/16	15/13	0.97
Age (years, mean ± SD)	34.50 ± 8.82	47.30 ± 11.10	41.00 ± 13.15	0.05
Median Age (years, range)	35.50 (20-52)	50 (18-60)	39 (18-60)	NA
TBSA (%, mean ± SD)	NA	58.18 ± 22.46	15.43 ± 4.90	NA
Median TBSA (%, range)	NA	50 (32-97)	15 (6-28)	NA
Time from admission to blood draw (h, mean ± SD)	NA	5.58 ± 2.81	5.64 ± 3.00	NA

### 3.2 Serum metabolomics analysis

**Figure 1:**
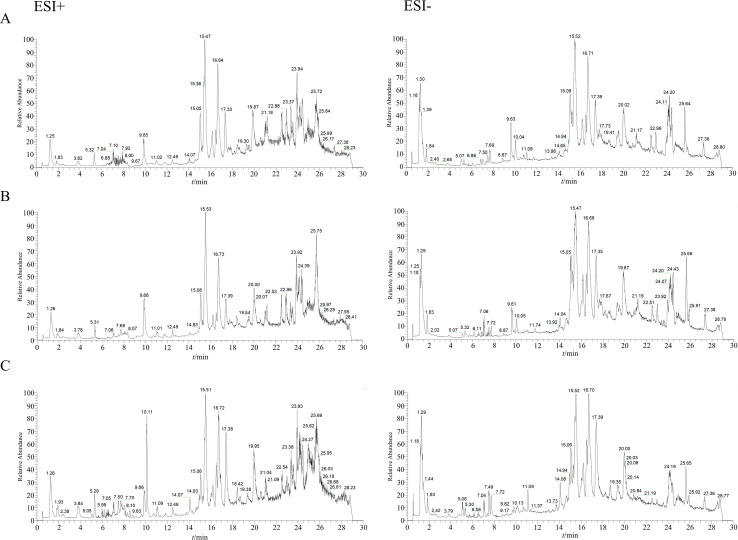
TICs of serum metabolites acquired under ESI+ and ESI− modes. Serum samples were obtained from healthy controls (n=18), burn patients with ≥30% TBSA (n=33), and burn patients with <30% TBSA (n=28). **(A)** Healthy controls; **(B)** Patients with ≥30% TBSA burns; **(C)** Patients with <30% TBSA burns.

**Figure 2:**
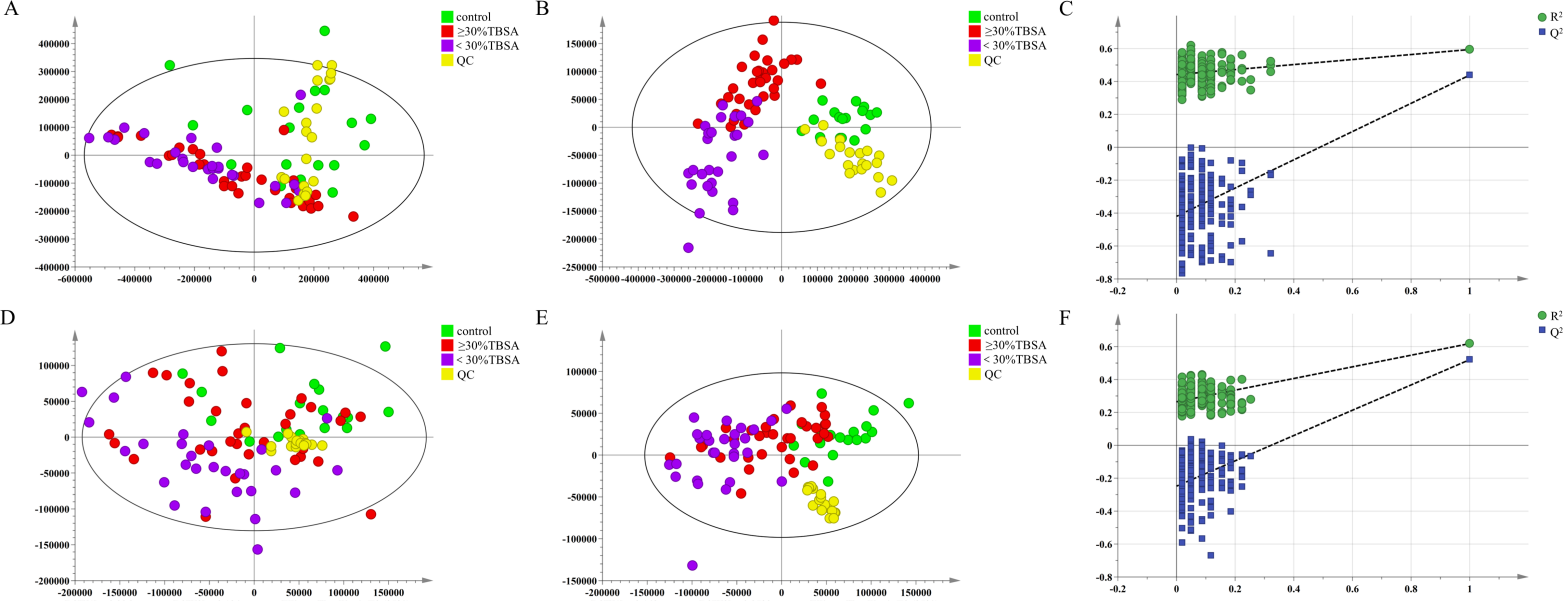
Multivariate statistical analysis of serum metabolomic profiles among study groups. Serum metabolomic data from healthy controls (n=18), burn patients with ≥30% TBSA (n=33), and burn patients with <30% TBSA (n=28) were analyzed using PCA and OPLS-DA. PCA was used as an unsupervised analysis method to evaluate global metabolic distribution patterns, whereas OPLS-DA was used as a supervised analysis method to assess intergroup metabolic separation. Each point represents an individual serum sample. Permutation testing was performed to evaluate model robustness and potential overfitting. In the permutation plots, *Q*^2^ values lower than *R*^2^ values and *Q*^2^ regression lines intersecting below zero indicated acceptable model validity. **(A)** PCA scores (ESI+); **(B)** OPLS-DA scores (ESI+); **(C)** Permutation test (ESI+); **(D)** PCA scores (ESI−); **(E)** OPLS-DA scores (ESI−); **(F)** Permutation test (ESI−).

Serum samples from healthy adults and burn patients with ≥30% TBSA and <30% TBSA were analyzed under the conditions described in Section 2.4. Total ion chromatograms (TICs) were obtained for each group. Results showed that low-molecular-weight endogenous metabolites (<1,200 Da) were successfully separated within 30 min. Variations in peak shapes and intensities among serum profiles indicated altered metabolic states and differential metabolite levels in burn patients (**Figure 1**). Metabolomics analysis identified 1,526 metabolites in ESI+ and 1,169 metabolites in ESI−. PCA and OPLS-DA analyses demonstrated tight clustering of QC samples, indicating high instrument stability and data reproducibility. In addition, pooled QC samples showed low analytical variation throughout the acquisition sequence, supporting the robustness of the LC-MS platform and the reliability of downstream metabolomics analyses. In both modes, healthy adults and burn patients were clearly separated, suggesting substantial metabolic alterations in the burn groups. In addition, a direct OPLS-DA comparison between the TBSA ≥30% and TBSA <30% groups demonstrated partial separation between groups, indicating the presence of severity-associated metabolic differences (**Supplementary Figure 1**). Permutation testing confirmed model validity, with *Q*^2^ (predictive ability) consistently lower than *R*^2^ (explained variance) values across all models. The *Q*^2^ regression line intersected the vertical axis below zero, confirming the absence of model overfitting and supporting robust interpretability and predictive capability (**Figure 2**).

### 3.3 Exploratory candidate biomarkers screening

**Figure 3:**
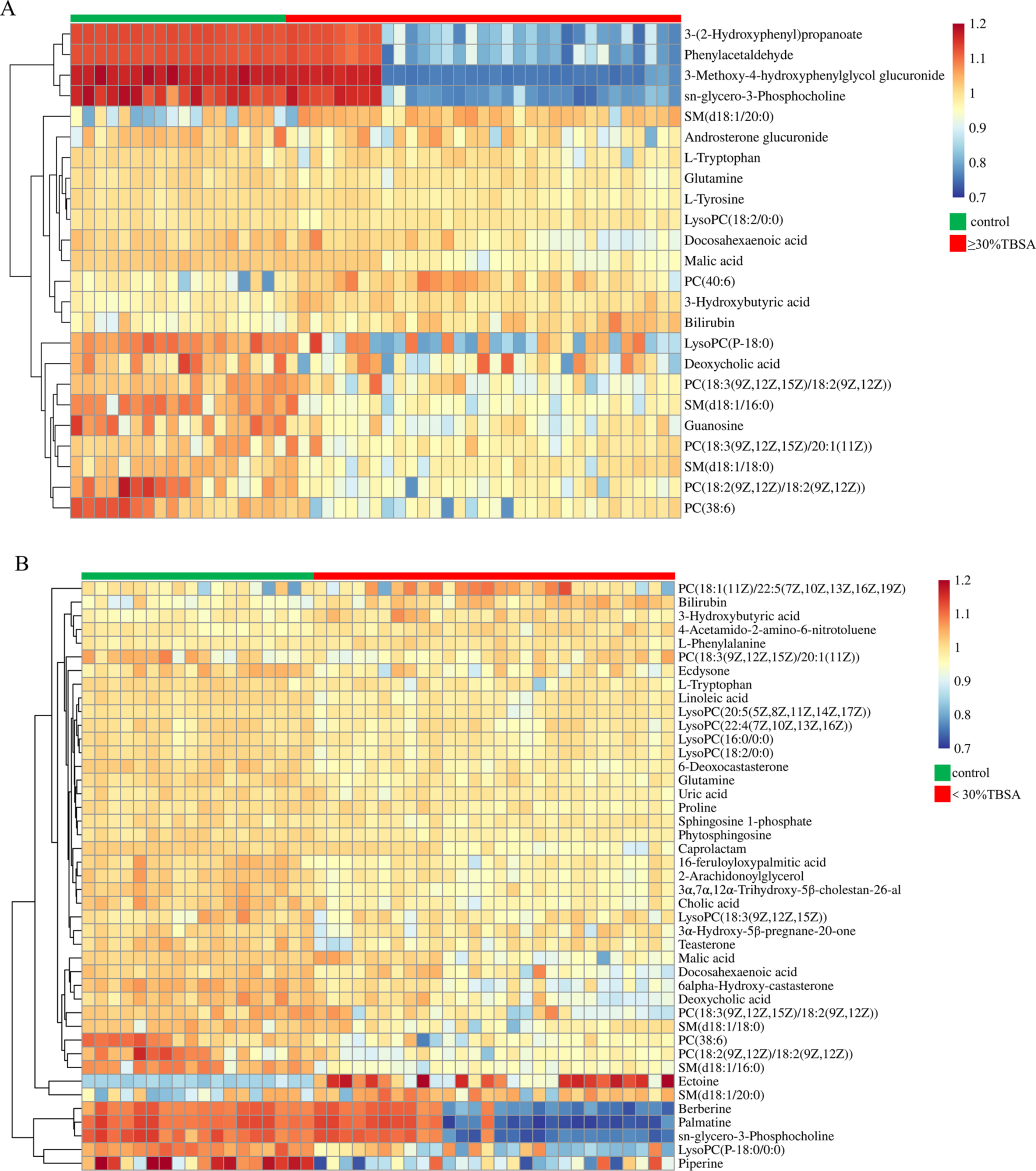
Heatmap analysis of exploratory candidate biomarkers identified by comparison with healthy controls. Heatmaps were generated using significantly altered metabolites identified between healthy controls (n=18) and burn patients with ≥30% TBSA (n=33) or <30% TBSA (n=28). Metabolite intensities were normalized, log-transformed, and visualized using hierarchical clustering analysis based on Euclidean distance and Ward’s linkage method. Each row represents an identified metabolite and each column represents an individual serum sample. The color scale represents z-score-normalized relative metabolite abundance across samples, with red indicating relatively higher abundance and blue indicating relatively lower abundance. Differential metabolites were screened based on VIP >1, *P* < 0.05, and FC >1.2 or <0.83. **(A)** Patients with ≥30% TBSA burns; **(B)** Patients with <30% TBSA burns.

Based on OPLS-DA analysis, differential metabolites were screened using VIP >1, *P* < 0.05, and FC >1.2 or <0.83. MS/MS spectra of selected metabolites were extracted and annotated against the HMDB and KEGG databases. Exploratory candidate biomarkers with unique KEGG IDs were further characterized based on KEGG annotation. Metabolite annotation was performed based on accurate mass, adduct pattern, isotope distribution, retention behavior, and MS/MS fragmentation similarity. According to MSI criteria, all reported metabolites were classified as MSI level 2 annotations. Compared with healthy controls, analysis identified 23 significantly altered exploratory candidate biomarkers in burn patients with ≥30% TBSA and 31 significantly altered exploratory candidate biomarkers in those with <30% TBSA (**Tables 2**, **3**). To further characterize severity-associated metabolic differences, the metabolic profiles of the TBSA ≥30% and TBSA <30% groups were additionally compared. The results demonstrated partially overlapping but distinct metabolite patterns between the two burn groups, supporting the presence of severity-related metabolic alterations. Hierarchical clustering heatmaps revealed metabolic profiles in each burn group that were distinct from those of healthy controls. These findings indicate a strong link between burn severity and metabolic dysregulation (**Figure 3**). Direct comparison between the TBSA ≥30% and TBSA <30% groups further identified metabolites associated with burn severity differences, as illustrated by volcano plot analysis (**Supplementary Figure 2**).

**Table 2:** Serum exploratory candidate biomarkers significantly altered in patients with ≥30% TBSA burns compared with healthy controls.

NO.	Metabolites	Ion mode	KEGG ID	HMDB ID	RT [min]	*m/z*	Formula	Reference Ion	MSI identification level	VIP	*P*	Adjust *P*	FC
1	SM (d18:1/20:0)	Positive	C00550	HMDB0012102	22.98	759.6369	C_43_H_87_N_2_O_6_P	[M+H]^+1^	Level 2	1.43	0.01	0.01	2.33
2	Bilirubin	Negative	C00486	HMDB0000054	9.66	583.2559	C_33_H_36_N_4_O_6_	[M-H]^-1^	Level 2	2.04	0.01	0.01	2.04
3	3-Hydroxybutyric acid	Negative	C01089	HMDB0000011	2.69	103.0402	C_4_H_8_O_3_	[M-H]^-1^	Level 2	4.21	0.01	0.01	1.79
4	LysoPC (18:2/0:0)	Positive	C04230	HMDB0010386	15.46	520.3392	C_26_H_50_NO_7_P	[M+H]^+1^	Level 2	10.02	0.01	0.01	0.82
5	L-Tryptophan	Positive	C00078	HMDB0000929	5.32	205.0972	C_11_H_12_N_2_O_2_	[M+H]^+1^	Level 2	1.28	0.02	0.05	0.77
6	Glutamine	Negative	C00064	HMDB0000641	1.28	145.0619	C_5_H_10_N_2_O_3_	[M-H]^-1^	Level 2	1.46	0.01	0.01	0.74
7	L-Tyrosine	Positive	C00082	HMDB0000158	1.94	182.0811	C_9_H_11_NO_3_	[M+H]^+1^	Level 2	1.61	0.01	0.01	0.71
8	Docosahexaenoic acid	Negative	C06429	HMDB0002183	20.4	327.2331	C_22_H_32_O_2_	[M-H]^-1^	Level 2	3.98	0.02	0.01	0.61
9	Androsterone glucuronide	Negative	C11135	HMDB0002829	10.09	465.2492	C_25_H_38_O_8_	[M-H]^-1^	Level 2	1.06	0.01	0.05	0.58
10	PC (18:3 (9Z,12Z,15Z)/20:1 (11Z))	Positive	C00157	HMDB0008209	22.54	810.6	C_46_H_84_NO_8_P	[M+H]^+1^	Level 2	3.31	0.04	0.01	0.56
11	Deoxycholic acid	Negative	C04483	HMDB0000626	12.94	391.2854	C_24_H_40_O_4_	[M-H]^-1^	Level 2	1.66	0.04	0.01	0.50
12	Malic acid	Negative	C00149	HMDB0000156	1.38	133.0143	C_4_H_6_O_5_	[M-H]^-1^	Level 2	2.41	0.01	0.01	0.50
13	SM (d18:1/18:0)	Positive	C00550	HMDB0001348	25.91	731.6061	C_41_H_83_N_2_O_6_P	[M+H]^+1^	Level 2	2.29	0.01	0.01	0.42
14	LysoPC (P-18:0)	Positive	C04230	HMDB0013122	21.16	508.3762	C_26_H_54_NO_6_P	[M+H]^+1^	Level 2	2.48	0.01	0.01	0.40
15	PC (18:3 (9Z,12Z,15Z)/18:2 (9Z,12Z))	Positive	C00157	HMDB0008204	24.76	780.5515	C_44_H_78_NO_8_P	[M+H]^+1^	Level 2	2.53	0.01	0.01	0.40
16	sn-glycero-3-Phosphocholine	Positive	C00670	HMDB0000086	7.69	336.123	C_8_H_21_NO_6_P	[M+DMSO+H]^+1^	Level 2	1.62	0.01	0.01	0.25
17	Phenylacetaldehyde	Negative	C00601	HMDB0006236	5.55	165.0557	C_8_H_8_O	[M+FA-H]^-1^	Level 2	1.03	0.01	0.01	0.24
18	Guanosine	Negative	C00387	HMDB0000133	2.05	282.0843	C_10_H_13_N_5_O_5_	[M-H]^-1^	Level 2	1.02	0.01	0.01	0.24
19	3- (2-Hydroxyphenyl)propanoate	Negative	C01198	HMDB0033752	5.55	165.0557	C_9_H_10_O_3_	[M-H]^-1^	Level 2	1.03	0.01	0.01	0.24
20	3-Methoxy-4-hydroxyphenylglycol glucuronide	Negative	C03033	HMDB0000496	4.02	359.0982	C_15_H_20_O_10_	[M-H]^-1^	Level 2	1.66	0.01	0.01	0.22
21	SM (d18:1/16:0)	Positive	C00550	HMDB0010169	22.98	703.5744	C_39_H_79_N_2_O_6_P	[M+H]^+1^	Level 2	10.89	0.01	0.01	0.13
22	PC (38:6)	Positive	C00157	HMDB0007991	22.99	806.5685	C_46_H_80_NO_8_P	[M+H]^+1^	Level 2	5.39	0.01	0.01	0.11
23	PC (18:2 (9Z,12Z)/18:2 (9Z,12Z))	Positive	C00157	HMDB0008138	21.1	782.5685	C_44_H_80_NO_8_P	[M+H]^+1^	Level 2	14.57	0.01	0.01	0.07

**Table 3:** Serum exploratory candidate biomarkers significantly altered in patients with <30% TBSA burns compared with healthy controls.

NO.	Metabolites	Ion Mode	KEGG ID	HMDB ID	RT [min]	*m/z*	Formula	Reference Ion	MSI Identification Level	VIP	*P*	adjust *P*	FC
1	Bilirubin	Negative	C00486	HMDB0000054	9.66	583.26	C_33_H_36_N_4_O_6_	[M-H]^-1^	Level 2	2.05	0.01	0.01	2.70
2	SM (d18:1/20:0)	Positive	C00550	HMDB0012102	22.98	759.64	C_43_H_87_N_2_O_6_P	[M+H]^+1^	Level 2	1.19	0.01	0.01	2.63
3	3-Hydroxybutyric acid	Negative	C01089	–	2.69	103.04	C_4_H_8_O_3_	[M-H]^-1^	Level 2	2.94	0.01	0.01	1.96
4	L-Phenylalanine	Negative	C00079	HMDB0000159	3.85	164.07	C_9_H_11_NO_2_	[M-H]^-1^	Level 2	1.12	0.01	0.02	1.27
5	LysoPC (20:5 (5Z,8Z,11Z,14Z,17Z))	Positive	C04230	HMDB0010397	15.53	542.32	C_28_H_48_NO_7_P	[M+H]^+1^	Level 2	1.33	0.01	0.01	0.82
6	Sphingosine 1-phosphate	Negative	C06124	HMDB0000277	13.36	378.24	C_18_H_38_NO_5_P	[M-H]^-1^	Level 2	1.62	0.01	0.05	0.76
7	Proline	Positive	C00148	HMDB0000162	1.35	116.07	C_5_H_9_NO_2_	[M+H]^+1^	Level 2	1.18	0.01	0.02	0.71
8	Uric acid	Negative	C00366	HMDB0000289	1.83	167.02	C_5_H_4_N_4_O_3_	[M-H]^-1^	Level 2	3.36	0.01	0.05	0.70
9	LysoPC (16:0/0:0)	Positive	C04230	HMDB0010382	16.12	496.34	C_24_H_50_NO_7_P	[M+H]^+1^	Level 2	6.61	0.01	0.01	0.70
10	L-Tryptophan	Positive	C00078	HMDB0000929	5.32	205.1	C_11_H_12_N_2_O_2_	[M+H]^+1^	Level 2	1.7	0.01	0.01	0.67
11	LysoPC (22:4 (7Z,10Z,13Z,16Z))	Positive	C04230	HMDB0010401	17.49	572.37	C_30_H_54_NO_7_P	[M+H]^+1^	Level 2	1.92	0.01	0.04	0.67
12	Linoleic acid	Negative	C01595	HMDB0000673	15.47	279.23	C_18_H_32_O_2_	[M-H]^-1^	Level 2	1.03	0.01	0.01	0.67
13	3α-Hydroxy-5β-pregnane-20-one	Positive	C05477	HMDB0006759	23.94	319.26	C_21_H_34_O_2_	[M+H]^+1^	Level 2	5.1	0.01	0.02	0.67
14	Phytosphingosine	Positive	C12144	HMDB0004610	11.1	318.3	C_18_H_39_NO_3_	[M+H]^+1^	Level 2	1.58	0.01	0.01	0.62
15	Docosahexaenoic acid	Negative	C06429	HMDB0002183	20.4	327.23	C_22_H_32_O_2_	[M-H]^-1^	Level 2	2.4	0.04	0.01	0.62
16	Malic acid	Negative	C00149	HMDB0000156	1.38	133.01	C_4_H_6_O_5_	[M-H]^-1^	Level 2	1.19	0.01	0.01	0.62
17	Glutamine	Negative	C00819	HMDB0003423	1.28	145.06	C_5_H_10_N_2_O_3_	[M-H]^-1^	Level 2	1.59	0.01	0.01	0.61
18	LysoPC (18:3 (9Z,12Z,15Z))	Positive	C04230	HMDB0010388	14.27	518.32	C_26_H_48_NO_7_P	[M+H]^+1^	Level 2	1.25	0.01	0.05	0.60
19	LysoPC (18:2/0:0)	Positive	C04230	HMDB0010386	15.04	520.34	C_26_H_50_NO_7_P	[M+H]^+1^	Level 2	9.62	0.01	0.01	0.60
20	PC (18:3 (9Z,12Z,15Z)/20:1 (11Z))	Positive	C00157	HMDB0008209	21.12	810.6	C_46_H_84_NO_8_P	[M+H]^+1^	Level 2	3.25	0.02	0.01	0.52
21	PC (18:3 (9Z,12Z,15Z)/18:2 (9Z,12Z))	Positive	C00157	HMDB0008204	24.76	780.55	C_44_H_78_NO_8_P	[M+H]^+1^	Level 2	2.05	0.01	0.01	0.48
22	SM (d18:1/18:0)	Positive	C00550	HMDB0001348	27.37	731.61	C_41_H_83_N_2_O_6_P	[M+H]^+1^	Level 2	1.12	0.01	0.01	0.47
23	3α,7α,12α-Trihydroxy-5β-cholestan-26-al	Negative	C01301	HMDB0003533	21.29	433.33	C_27_H_46_O_4_	[M-H]^-1^	Level 2	1.04	0.01	0.01	0.45
24	2-Arachidonoylglycerol	Negative	C13856	HMDB0004666	18.92	377.27	C_23_H_38_O_4_	[M-H]^-1^	Level 2	1.18	0.01	0.01	0.44
25	Deoxycholic acid	Negative	C04483	HMDB0000626	20.4	391.29	C_24_H_40_O_4_	[M-H]^-1^	Level 2	2.05	0.01	0.01	0.41
26	Cholic acid	Negative	C00695	HMDB0000619	18.73	407.28	C_24_H_40_O_5_	[M-H]^-1^	Level 2	1.05	0.01	0.01	0.40
27	sn-glycero-3-Phosphocholine	Positive	C00670	HMDB0000086	7.69	337.12	C_8_H_21_NO_6_P	[M+DMSO+H]^+1^	Level 2	1.09	0.01	0.01	0.37
28	LysoPC (P-18:0/0:0)	Positive	C04230	HMDB0013122	21.16	508.38	C_26_H_54_NO_6_P	[M+H]^+1^	Level 2	2.97	0.01	0.01	0.24
29	PC (38:6)	Positive	C00157	HMDB0007991	22.99	806.57	C_46_H_80_NO_8_P	[M+H]^+1^	Level 2	4.08	0.01	0.01	0.14
30	SM (d18:1/16:0)	Positive	C00550	HMDB0010169	22.98	703.57	C_39_H_79_N_2_O_6_P	[M+H]^+1^	Level 2	8.53	0.01	0.01	0.10
31	PC (18:2 (9Z,12Z)/18:2 (9Z,12Z))	Positive	C00157	HMDB0008138	21.1	782.57	C_44_H_80_NO_8_P	[M+H]^+1^	Level 2	8.86	0.01	0.01	0.09

### 3.4 Metabolic pathway enrichment analysis

**Figure 4:**
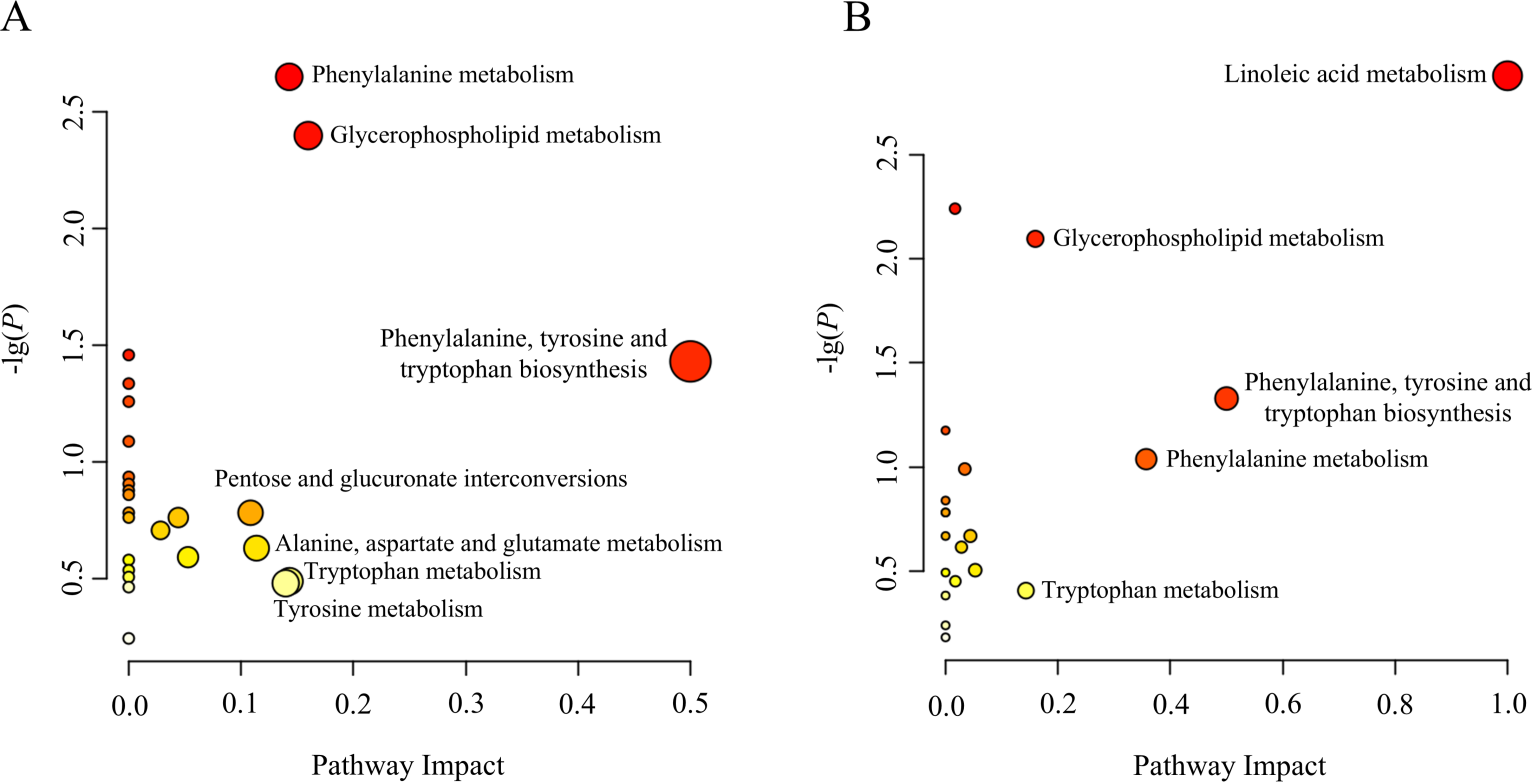
Metabolic pathway enrichment analysis of exploratory candidate biomarkers in burn patients. Pathway enrichment analysis was performed using the KEGG database through the MetaboAnalyst platform based on significantly altered metabolites identified in each burn group compared with healthy controls. The y-axis represents enriched metabolic pathways, and the x-axis represents pathway impact values. Bubble size indicates the number of enriched differential metabolites within each pathway, whereas bubble color reflects pathway enrichment significance, with darker colors indicating higher statistical significance. Pathways with *P* < 0.05 were considered significantly enriched. **(A)** Patients with ≥30% TBSA burns; **(B)** Patients with <30% TBSA burns.

To further elucidate the functional pathways implicated by exploratory candidate biomarkers, metabolic pathway enrichment analysis was performed using the MetaboAnalyst platform. Functional networks among metabolites were then constructed. Based on comparisons between each burn group and healthy controls, analysis revealed marked differences in pathway dysregulation among patients with different burn severities. Patients with severe burns exhibited broader disturbances in amino acid metabolism and energy-related pathways, whereas patients with smaller burns showed relatively concentrated alterations in lipid-related metabolic pathways. In the ≥30% TBSA burn group, 7 significantly altered pathways were identified. These included phenylalanine, tyrosine, and tryptophan biosynthesis; phenylalanine metabolism; glycerophospholipid metabolism; alanine, aspartate, and glutamate metabolism; tyrosine metabolism; pentose and glucuronate interconversions; and tryptophan metabolism. In the <30% TBSA burn group, 5 altered pathways were detected. They primarily involved linoleic acid metabolism; glycerophospholipid metabolism; phenylalanine, tyrosine, and tryptophan biosynthesis; phenylalanine metabolism; and tryptophan metabolism (**Figure 4**). Patients with more extensive burns in this cohort exhibited alterations in a broader range of metabolic pathways compared with patients with smaller burns.

**Figure 5:**
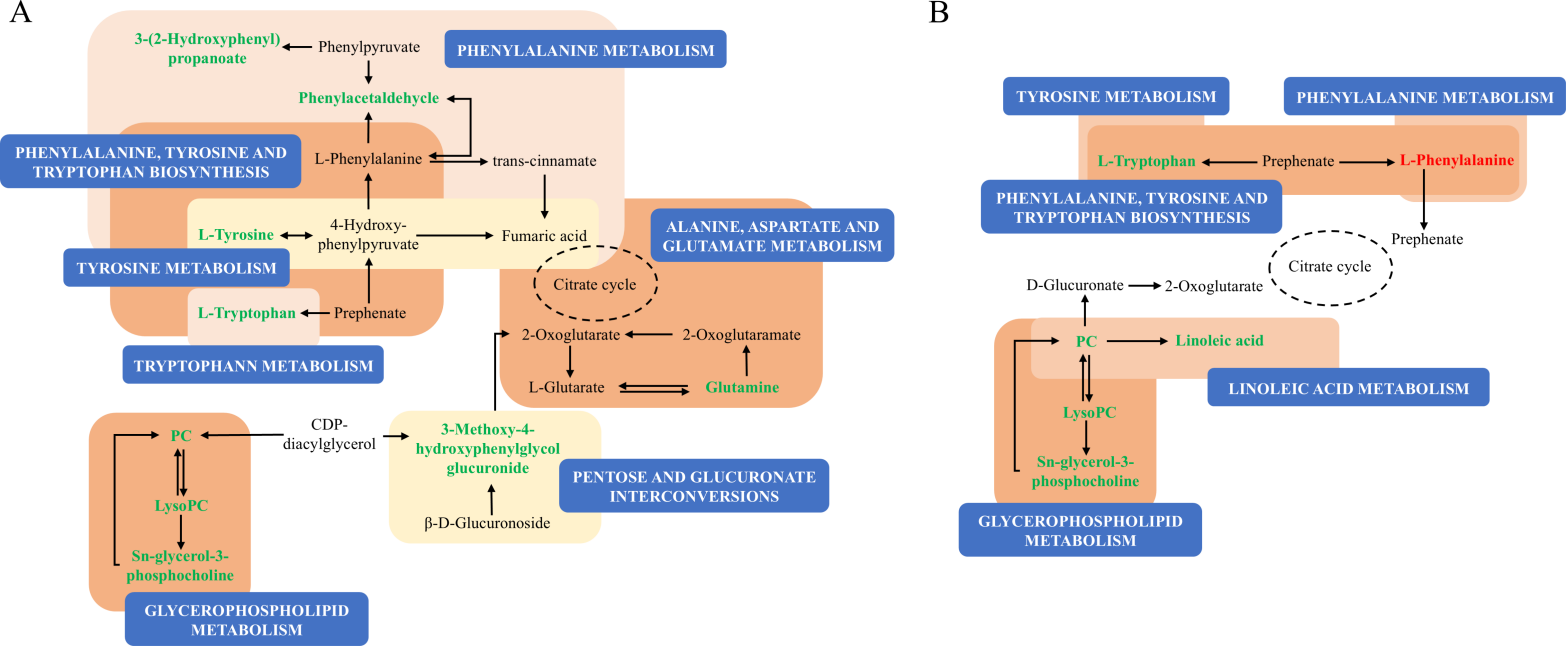
Metabolic pathway network analysis of exploratory candidate biomarkers in burn patients. Metabolic interaction networks were constructed using KEGG pathway analysis based on significantly altered metabolites identified in each burn group compared with healthy controls. Red nodes indicate relatively increased-abundance metabolites, whereas green nodes indicate relatively decreased-abundance metabolites. **(A)** Patients with ≥30% TBSA burns; **(B)** Patients with <30% TBSA burns.

**Figure 6:**
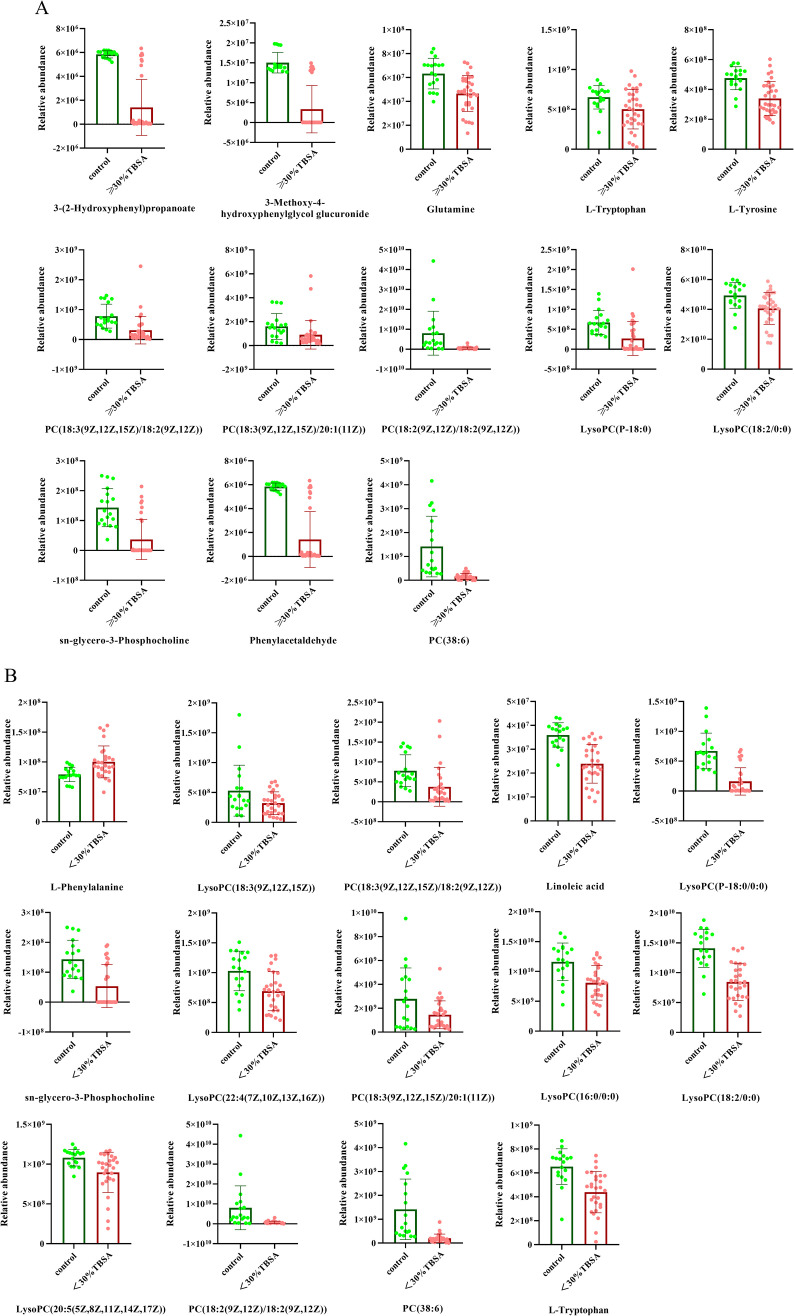
Relative abundance profiles of burn-related exploratory candidate biomarkers. The figure displays relative abundance differences of significantly altered exploratory candidate biomarkers identified in burn patients compared with healthy controls. **(A)** Patients with ≥30% TBSA burns; **(B)** Patients with <30% TBSA burns.

Using KEGG tools, a metabolic interaction network was constructed to visualize pathway alterations (**Figure 5**). In patients with ≥30% TBSA burns, seven significantly altered metabolic pathways were identified, involving 13 burn-related exploratory candidate biomarkers. In patients with <30% TBSA burns, five disrupted pathways were observed, involving 14 burn-related exploratory candidate biomarkers. The relative abundance of these exploratory candidate biomarkers is shown in **Figure 6**.

### 3.5 ROC analysis of exploratory candidate biomarkers in key metabolic pathways

**Figure 7:**
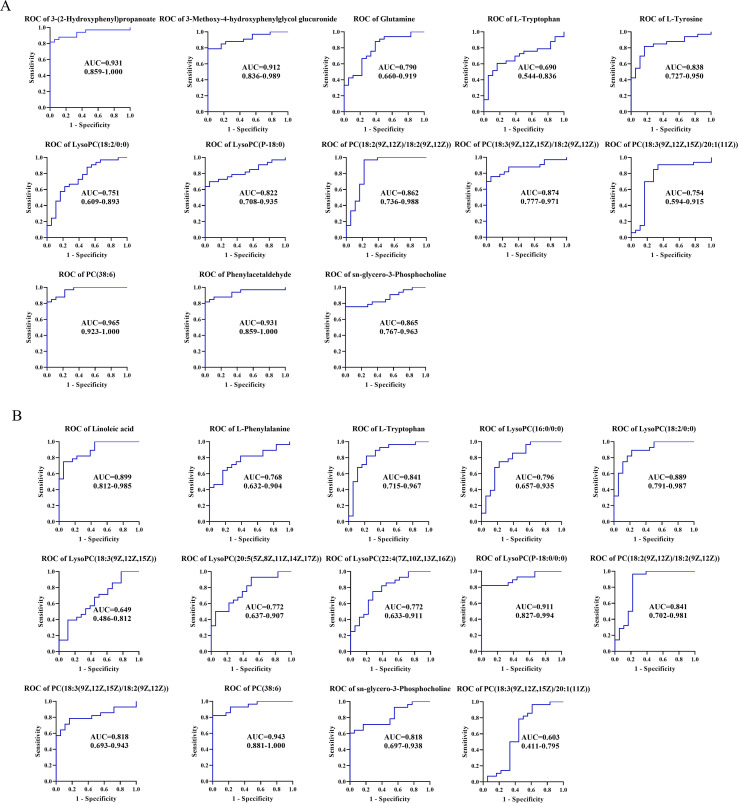
ROC analysis of exploratory candidate biomarkers associated with burn severity. ROC analysis was performed using metabolite intensities from the discovery cohort to preliminarily evaluate the discriminatory performance of selected exploratory candidate biomarkers between burn patients and healthy controls. The x-axis represents 1-specificity, and the y-axis represents sensitivity. The AUC was calculated for each metabolite. No internal cross-validation or independent external validation cohort was applied; therefore, these results should be interpreted as exploratory findings within the current cohort. Metabolites with an AUC >0.8 were considered to demonstrate potential discriminatory performance. AUC values, 95% confidence intervals, and specificity values are indicated in the figure. **(A)** Patients with ≥30% TBSA burns; **(B)** Patients with <30% TBSA burns.

To preliminarily evaluate the discriminatory performance of exploratory candidate biomarkers in key metabolic pathways, ROC analysis was performed (**Figure 7**). In patients with ≥30% TBSA burns, nine exploratory candidate biomarkers demonstrated potential discriminatory performance with an AUC >0.8. These exploratory candidate biomarkers included 3-(2-Hydroxyphenyl) propanoate, 3-Methoxy-4-hydroxyphenylglycol glucuronide, L-Tyrosine, LysoPC (P-18:0), PC [18:2(9Z,12Z)/18:2(9Z,12Z)], PC [18:3(9Z,12Z,15Z)/18:2(9Z,12Z)], PC (38:6), Phenylacetaldehyde, and sn-glycero-3-Phosphocholine. These exploratory candidate biomarkers were mainly involved in phenylalanine metabolism, pentose and glucuronate interconversions, tyrosine metabolism, and glycerophospholipid metabolism. In patients with <30% TBSA burns, eight exploratory candidate biomarkers demonstrated potential discriminatory performance with an AUC >0.8 within the current cohort. These exploratory candidate biomarkers included Linoleic acid, L-Tryptophan, LysoPC (18:2/0:0), LysoPC (P-18:0/0:0), PC (18:2(9Z,12Z)/18:2(9Z,12Z)), PC (18:3(9Z,12Z,15Z)/18:2(9Z,12Z)), PC (38:6), and sn-glycero-3-Phosphocholine. These exploratory candidate biomarkers were primarily involved in linoleic acid metabolism, tryptophan metabolism, and glycerophospholipid metabolism.

## 4 Discussion

Burn injury represents a major global public health challenge, with burn-induced metabolic disorders being among the most critical complications that substantially influence both short-term recovery and long-term prognosis (Ryan et al., 2024). After a burn, patients develop multiple interconnected metabolic abnormalities. These abnormalities form a complex pathophysiological network, including progressive muscle wasting, sustained hypermetabolism, persistent hyperglycemia, elevated lactate, insulin resistance, and mitochondrial dysfunction (Alkhalil et al., 2021; Yang et al., 2022; Gao et al., 2025; Wei et al., 2025). Metabolomics has emerged as an effective tool for characterizing these metabolic changes in burn patients and for identifying clinically relevant biomarkers (Elmassry et al., 2020; Nakazawa et al., 2022). In this study, we systematically analyzed the serum metabolic profiles of patients with burns covering ≥30% and <30% TBSA using UPLC-MS/MS. We found that burns of different severities were associated with marked metabolic disturbances, which vary in their magnitude, affected pathways, and exploratory candidate biomarkers associated with burn severity-related metabolic alterations. Importantly, the identified exploratory candidate biomarkers were primarily derived from separate comparisons of each burn group with healthy controls, while severity-associated exploratory metabolic signatures were inferred through comparative interpretation of the two burn groups. Direct comparison between TBSA ≥30% and TBSA <30% groups further supported distinct metabolic trends associated with burn severity.

### 4.1 Amino acid metabolism

Amino acid metabolism was markedly altered in both burn groups. Among these, the aromatic amino acids—phenylalanine, tyrosine, and tryptophan—are closely associated with burn-related metabolic disturbances. Phenylalanine is an essential amino acid that often shows increased serum levels after burn injury. This rise may reflect burn-associated hypermetabolism, stress responses, and impaired hepatic metabolic regulation, consistent with previous reports (Sommerhalder et al., 2020; Knuth et al., 2021; Su et al., 2024). Previous studies have suggested that burns frequently induce SIRS, during which inflammatory responses may suppress phenylalanine hydroxylase (PAH) activity and reduce conversion efficiency from phenylalanine to tyrosine. This process may contribute to phenylalanine accumulation following burn injury (Begum et al., 2023; She et al., 2023). Tyrosine, a downstream product of phenylalanine, often shows reduced levels in burn patients. This decline likely reflects the direct consequence of PAH inhibition. Decreased tyrosine not only limits its role as a precursor for neurotransmitter synthesis but may also indirectly disturb tryptophan metabolism (Strasser et al., 2017; Giret et al., 2023). Tryptophan is an essential amino acid with critical roles in the nervous, endocrine, and immune systems through its metabolic pathways (Tsuji et al., 2023). Studies indicate that tryptophan metabolism is strongly linked to inflammation and inflammatory diseases. As a precursor to immunomodulatory molecules, tryptophan may be extensively consumed under burn-associated hypermetabolism, inflammation, and oxidative stress, according to previous studies (Gao et al., 2025). Notably, tryptophan may undergo accelerated catabolism via the kynurenine pathway, a process previously reported to be associated with immunosuppression and impaired tissue repair capacity (Ploder et al., 2010; Molfino et al., 2024).

Additionally, metabolic products of certain aromatic amino acids show significant abnormalities in burn patients. 3-(2-Hydroxyphenyl) propanoate, a metabolite derived from tyrosine via microbial or host enzyme catalysis, exhibits antioxidant and anti-inflammatory activities (Strauss et al., 2023). Phenylacetaldehyde, a metabolite generated from phenylalanine through deamination of the phenethylamine intermediate, also demonstrates anti-inflammatory effects (Lee et al., 2025). Both metabolites showed significantly decreased serum levels, which may reflect impaired antioxidant defense and disrupted immune regulation following burn injury. In this study, serum levels of L-Tryptophan, L-Tyrosine, 3-(2-Hydroxyphenyl) propanoate, and Phenylacetaldehyde were significantly altered in patients with burns covering ≥30% TBSA. In patients with burns covering <30% TBSA, only L-Phenylalanine and L-Tryptophan showed significant changes. These findings suggest distinct alterations in aromatic amino acids and their metabolites according to burn severity. Patients with ≥30% TBSA burns exhibited more complex and widespread metabolic disturbances, which may reflect greater systemic stress and immune dysregulation.

### 4.2 Glycerophospholipid metabolism

Following burns, the body undergoes complex metabolic reprogramming, with altered glycerophospholipid metabolism being commonly observed after burn injury. Glycerophospholipids, including phosphatidylcholine (PCs), phosphatidylethanolamine (PEs), and lysophosphatidylcholine (LysoPCs), are essential components of cell membranes. As structural elements and sources of signaling molecules, dynamic alterations in glycerophospholipid metabolism can significantly influence post-burn pathophysiology (Kierath et al., 2023). LysoPCs represent a crucial class of glycerophospholipid metabolites with dual roles in immune regulation, they induce chemotaxis and participate in early immune activation. Sustained serum downregulation of LysoPCs observed in this study may be associated with altered inflammatory and immune responses after burns, as suggested by previous studies (Engel et al., 2021). Previous studies have shown that hypoalbuminemia (albumin concentration <20 g/L) correlates with reduced blood LysoPCs levels. Hypoalbuminemia is common in sepsis, burns, and severe trauma (Sun et al., 2015) and may partially explain the diminished LysoPCs levels observed in burn patients. PCs are key membrane lipids that maintain membrane fluidity and integrity and play crucial roles in membrane repair, signal transduction, and inflammatory responses. Studies indicate that PC depletion can impair cell membrane repair, disrupt signaling pathways, and promote abnormal release of proinflammatory mediators (Cauvi et al., 2024). Pashkevich et al (Pashkevich et al., 2024) further demonstrated this in a rat model of II-IIIA degree thermal burns. Administration of PC-enriched liposomes significantly improved the structure and barrier function of burned skin and effectively suppressed inflammatory responses and secondary tissue damage. Furthermore, sn-glycerol-3-Phosphocholine, an intermediate metabolite in glycerophospholipid metabolism involved in phospholipid synthesis and choline metabolism, shows decreased serum levels in burn patients. This decline may indicate impaired phospholipid synthesis capacity, further highlighting disruption of cell membrane structural integrity and sustained release of inflammatory mediators following burns (Laserna et al., 2020). In our study, serum levels of LysoPCs, PCs, and sn-glycero-3-Phosphocholine were significantly decreased in patients with burns covering both ≥30% and <30% of TBSA. This finding suggests that even mild burns may be associated with impaired membrane stability and disrupted glycerophospholipid metabolism. The altered metabolic profile may reflect inflammatory and stress responses following burn injury. It may also be closely associated with reduced membrane structural integrity, impaired signal transduction, and dysregulated immune function.

### 4.3 Linoleic acid metabolism

In patients with burns covering <30% TBSA, plasma linoleic acid and PC levels were significantly decreased, indicating altered in linoleic acid metabolism following burns. Linoleic acid, an omega-6 fatty acid, is a crucial component of human tissues. Studies indicate that under severe stress conditions such as burns, its oxidative metabolites can profoundly affect immune cell function and drive pathological inflammatory responses (Bergmann et al., 2022; Mercola and D'Adamo, 2023). During the early phase of burn injury, the body experiences intense stress with markedly enhanced fat mobilization. Previous studies have suggested that severe stress responses after burns are associated with enhanced adipose tissue lipolysis and increased fatty acid mobilization into the circulation (Barayan et al., 2020; Kaur et al., 2020). Linoleic acid is rapidly consumed in this process due to its roles in cellular repair, signal transduction, and inflammatory regulation, which likely accounts for its decreased serum levels. Interestingly, no significant disruption in linoleic acid metabolism was observed in patients with burns covering ≥30% TBSA. This may potentially be associated with altered hepatic metabolic capacity affecting linoleic acid turnover in patients with ≥30% TBSA burns (Song et al., 2014; Gong et al., 2018; Yang et al., 2022). These factors may limit further metabolic conversion of linoleic acid, resulting in relatively stable serum levels.

### 4.4 Alanine, aspartate, and glutamate metabolism

This study found that the alanine, aspartate, and glutamate metabolism pathway was markedly altered in patients with burns covering ≥30% TBSA. Glutamine is closely involved in this pathway and serves as an essential nutritional substrate. Under stress conditions such as burns or burn sepsis, glutamine supports multiple cellular functions and maintains metabolic homeostasis (Wischmeyer, 2008; Moreira et al., 2018; Wischmeyer, 2019). Previous studies have shown that glutamine repairs impaired mitochondrial electron transport chain (ETC) complexes, thereby potentially supporting energy metabolism. It also reduces oxidative stress and protects hepatocytes from burn-related sepsis injury (Yang et al., 2024). In addition, glutamine facilitates macrophage M2 polarization by inhibiting SIRT5-mediated desuccinylation of pyruvate dehydrogenase. It also attenuates burn sepsis and liver injury by promoting SIRT4-dependent HSP60-HSP10 complex assembly, which has been reported to support mitochondrial energy metabolism (Zhu et al., 2022; Yang et al., 2024). In this study, serum glutamine levels were markedly reduced in patients with burns covering ≥30% TBSA. This reduction may be associated with systemic hypermetabolism and inflammation following extensive burns, which have been reported to accelerate muscle protein breakdown. At the same time, increased glutamine uptake and consumption by the liver and immune system may further deplete circulating levels. In addition, wound exudate causes substantial loss of soluble amino acids, including glutamine and alanine. This loss has been reported to be positively correlated with burn area (Berger et al., 2022).

### 4.5 Pentose and glucuronate interconversions

Studies have shown that the pentose and glucuronate interconversions pathway is disrupted in patients with ≥30% TBSA severe burns. Serum levels of 3-Methoxy-4-hydroxyphenylglycol glucuronide (MHPG-glucuronide) are significantly reduced in these patients. This pathway is involved in pentose metabolism and in the production of UDP-glucuronic acid (UDP-GlcA). It also plays a critical role in hepatic glucuronidation and detoxification reactions (TeSlaa et al., 2023). Multiple studies indicate that severe burns induce strong inflammatory responses and oxidative stress. These changes may enhance pentose phosphate pathway activity to meet the increased demand for NADPH during oxidative stress. However, this shift may reduce UDP-GlcA availability and ultimately impairs hepatic glucuronidation of catecholamine metabolites, which could potentially contribute to lower serum MHPG-glucuronide levels (Rehou et al., 2023; Su et al., 2024; Wei et al., 2025). As the major conjugated metabolite of norepinephrine, reduced MHPG-glucuronide may be associated with altered catecholamine-related metabolic processing and changes in hepatic conjugation-related metabolism (Lelou et al., 2022). In contrast, patients with <30% TBSA burns did not show this metabolic abnormality, possibly due to milder traumatic stress and relatively preserved hepatic blood flow and UGT activity.

Finally, ROC analysis identified several exploratory candidate biomarkers with potential discriminatory performance and suggested their potential association with burn severity. Using AUC >0.8 as the threshold, nine exploratory candidate biomarkers were identified in patients with burns covering ≥30% TBSA. These included 3-(2-Hydroxyphenyl) propanoate, 3-Methoxy-4-hydroxyphenylglycol glucuronide, L-Tyrosine, LysoPC (P-18:0), PC (18:2(9Z,12Z)/18:2(9Z,12Z)), PC (18:3(9Z,12Z,15Z)/18:2(9Z,12Z)), PC (38:6), Phenylacetaldehyde, and sn-glycero-3-Phosphocholine. These exploratory candidate biomarkers were mainly associated with pathways related to phenylalanine metabolism, tyrosine metabolism, pentose and glucuronate interconversions, and glycerophospholipid metabolism. In patients with burns involving <30% TBSA, several metabolites were identified as exploratory candidate biomarkers associated with burn severity-related metabolic alterations. These included Linoleic acid, L-Tryptophan, LysoPC (18:2/0:0), LysoPC (P-18:0/0:0), PC (18:2(9Z,12Z)/18:2(9Z,12Z)), PC (18:3(9Z,12Z,15Z)/18:2(9Z,12Z)), PC (38:6), and sn-glycero-3-Phosphocholine. They were primarily associated with linoleic acid metabolism, tryptophan metabolism, and glycerophospholipid metabolism pathways. A higher AUC value suggests potential discriminatory capability for differentiating metabolic characteristics associated with different burn severities within the current cohort. Clinically, the identified exploratory candidate biomarkers and pathways may represent candidates for future burn severity stratification research; prospective studies and mechanistic validation are needed before their potential clinical utility can be further evaluated. Furthermore, the altered amino acid and glycerophospholipid metabolism pathways may warrant further investigation. Future nutritional or pharmacological studies could explore their potential to modulate metabolic homeostasis, reduce systemic inflammation, and improve metabolic recovery.

## 5 Conclusions

This study suggests that patients with different burn severities exhibit distinct metabolic alterations. These findings were primarily based on comparisons between each burn group and healthy controls, which collectively revealed severity-associated metabolic patterns. Patients with burns covering <30% TBSA showed abnormalities in aromatic amino acid, glycerophospholipid, and linoleic acid metabolism. However, overall changes were relatively limited, potentially reflecting early inflammatory responses, membrane structural disruption, and metabolic mobilization. In contrast, patients with burns covering ≥30% TBSA exhibited more extensive and complex metabolic This study suggests that patients with different burn severities exhibit distinct metabolic alterations. This involved multiple key pathways, including aromatic amino acid, glycerophospholipid, alanine, aspartate, and glutamate metabolism, as well as pentose and glucuronate interconversions. These metabolic alterations may be associated with immune-related metabolic alterations, membrane structural disruption, systemic hypermetabolic responses, and impaired liver function, based on integration with previous experimental and clinical studies.

ROC analysis identified several exploratory candidate biomarkers with potential discriminatory performance (AUC >0.8) across different burn severity groups within the current cohort. These exploratory candidate biomarkers may provide preliminary metabolomics-based information for future burn severity stratification research. In particular, the identified biomarkers may represent candidate indicators associated with burn severity-related metabolic alterations, while the disrupted metabolic pathways may inform the design of targeted nutritional and pharmacological interventions.

Despite these findings, several limitations should be acknowledged. The relatively small sample size and single-center design may limit the generalizability of the results. Serum samples were collected only during the acute admission phase, and longitudinal sampling was not performed. In addition, although standardized sampling procedures and strict exclusion criteria were applied, residual confounding from clinical variables such as burn depth, nutritional status, infection, organ dysfunction, and therapeutic interventions could not be completely excluded. Furthermore, ROC analyses were conducted using the same cohort employed for exploratory candidate biomarkers discovery, and independent external validation was not performed due to the limited sample size. Therefore, the identified exploratory candidate biomarkers should be considered preliminary findings requiring further validation. Future studies should include larger, multicenter cohorts with longitudinal sampling to further validate the identified metabolic alterations and assess their clinical relevance. Integrating metabolomics with other omics approaches, such as proteomics and transcriptomics, may further improve our understanding of the systemic responses to burn injury.

## Data Availability

The original contributions presented in the study are included in the article/**Supplementary Material**. Further inquiries can be directed to the corresponding authors.

## Supplementary material

Supplementary Figure 1 Direct multivariate comparison between burn patients with ≥30% TBSA (n=33) and <30% TBSA (n=28). PCA and OPLS-DA analyses were performed to evaluate severity-associated metabolic differences between the two burn groups under both ESI+ and ESI− modes. PCA was used for unsupervised clustering analysis, whereas OPLS-DA was applied for supervised discrimination analysis. Each point represents an individual serum sample. Permutation testing was performed to assess model stability and potential overfitting. **(A)** PCA scores (ESI+); **(B)** OPLS-DA scores (ESI+); **(C)** Permutation test (ESI+); **(D)** PCA scores (ESI−); **(E)** OPLS-DA scores (ESI−); **(F)** Permutation test (ESI−).

Supplementary Figure 2 Volcano plot of differential metabolites between TBSA ≥30% and TBSA <30% burn groups. Differential metabolites were screened based on VIP >1, *P* < 0.05, and FC >1.2 or <0.83. Red dots indicate metabolites with significantly increased abundance, green dots indicate metabolites with significantly decreased abundance, and black dots represent non-significant metabolites. **(A)** ESI+; **(B)** ESI−.

## References

[B1] Alkhalil A. Ball R. L. Garg G. Day A. Carney B. C. Kumar R. . (2021). Cutaneous thermal injury modulates blood and skin metabolomes differently in a murine model. J. Burn Care Res. 42, 727–742. doi: 10.1093/jbcr/iraa209 33301570 PMC8335952

[B2] Barayan D. Vinaik R. Auger C. Knuth C. M. Abdullahi A. Jeschke M. G. (2020). Inhibition of lipolysis with acipimox attenuates postburn white adipose tissue browning and hepatic fat infiltration. Shock 53, 137–145. doi: 10.1097/shk.0000000000001439 31425403 PMC10880813

[B3] Begum S. Johnson B. Z. Morillon A. C. Yang R. Bong S. H. Whiley L. . (2022). Systemic long-term metabolic effects of acute non-severe paediatric burn injury. Sci. Rep. 12, 13043. doi: 10.1038/s41598-022-16886-w 35906249 PMC9338081

[B4] Begum S. Lodge S. Hall D. Johnson B. Z. Bong S. H. Whiley L. . (2023). Cardiometabolic disease risk markers are increased following burn injury in children. Front. Public Health 11, 1105163. doi: 10.3389/fpubh.2023.1105163 37333522 PMC10275366

[B5] Berger M. M. Binz P. A. Roux C. Charrière M. Scaletta C. Raffoul W. . (2022). Exudative glutamine losses contribute to high needs after burn injury. JPEN J. Parenter Enteral Nutr. 46, 782–788. doi: 10.1002/jpen.2227 34288001 PMC9292800

[B6] Bergmann C. B. McReynolds C. B. Wan D. Singh N. Goetzman H. Caldwell C. C. . (2022). sEH-derived metabolites of linoleic acid drive pathologic inflammation while impairing key innate immune cell function in burn injury. Proc. Natl. Acad. Sci. U.S.A. 119, e2120691119. doi: 10.1073/pnas.2120691119 35312372 PMC9060469

[B7] Brusselaers N. Monstrey S. Vogelaers D. Hoste E. Blot S. (2010). Severe burn injury in Europe: a systematic review of the incidence, etiology, morbidity, and mortality. Crit. Care 14, R188. doi: 10.1186/cc9300 20958968 PMC3219295

[B8] Cauvi D. M. Hawisher D. Derunes J. Rodriguez E. De Maio A. (2024). Membrane phospholipids activate the inflammatory response in macrophages by various mechanisms. FASEB J. 38, e23619. doi: 10.1096/fj.202302471r 38661031

[B9] Elmassry M. M. Mudaliar N. S. Colmer-Hamood J. A. San Francisco M. J. Griswold J. A. Dissanaike S. . (2020). New markers for sepsis caused by Pseudomonas aeruginosa during burn infection. Metabolomics 16, 40. doi: 10.1007/s11306-020-01658-2 32170472 PMC7223005

[B10] Engel K. M. Schiller J. Galuska C. E. Fuchs B. (2021). Phospholipases and reactive oxygen species derived lipid biomarkers in healthy and diseased humans and animals - a focus on lysophosphatidylcholine. Front. Physiol. 12, 732319. doi: 10.3389/fphys.2021.732319 34858200 PMC8631503

[B11] Gao S. Leng Y. Qiu Z. Li K. Li J. Peng J. . (2025). Burn-induced gut microbiota dysbiosis aggravates skeletal muscle atrophy by tryptophan-kynurenine mediated AHR pathway activation. Adv. Sci. (Weinh) 12, e2409296. doi: 10.1002/advs.202409296 39950940 PMC11984878

[B12] Giret C. Dos Santos Y. Blasco H. Paget C. Gonzalez L. Tressel N. . (2023). No evidence for systemic low-grade inflammation in adult patients with early-treated phenylketonuria: the INGRAPH study. JIMD Rep. 64, 446–452. doi: 10.1002/jmd2.12366 37927482 PMC10623104

[B13] Gong Y. Long X. Xu H. Yang X. Guo Q. (2018). The changes and prognostic value of liver function in young adults with severe burn: a retrospective observational study. Med. (Baltimore) 97, e13721. doi: 10.1097/md.0000000000013721 30572508 PMC6320172

[B14] Gu X. Dong Y. Wang X. Ren Z. Li G. Hao Y. . (2024). Identification of serum biomarkers for chronic kidney disease using serum metabolomics. Ren Fail. 46, 2409346. doi: 10.1080/0886022x.2024.2409346 39378112 PMC11463012

[B15] Jeschke M. G. van Baar M. E. Choudhry M. A. Chung K. K. Gibran N. S. Logsetty S. (2020). Burn injury. Nat. Rev. Dis. Primers 6, 11. doi: 10.1007/978-3-7091-1133-8_2 32054846 PMC7224101

[B16] Kaur S. Auger C. Jeschke M. G. (2020). Adipose tissue metabolic function and dysfunction: impact of burn injury. Front. Cell Dev. Biol. 8, 599576. doi: 10.3389/fcell.2020.599576 33251224 PMC7676399

[B17] Kierath E. Ryan M. Holmes E. Nicholson J. K. Fear M. W. Wood F. M. . (2023). Plasma lipidomics reveal systemic changes persistent throughout early life following a childhood burn injury. Burns Trauma 11, tkad044. doi: 10.1093/burnst/tkad044 38074192 PMC10703495

[B18] Knuth C. M. Auger C. Jeschke M. G. (2021). Burn-induced hypermetabolism and skeletal muscle dysfunction. Am. J. Physiol. Cell Physiol. 321, C58–c71. doi: 10.1152/ajpcell.00106.2021 33909503 PMC8321793

[B19] Laserna A. K. C. Lai Y. Fang G. Ganapathy R. Atan M. Lu J. . (2020). Metabolic profiling of a porcine combat trauma-injury model using NMR and multi-mode LC-MS metabolomics-a preliminary study. Metabolites 10. doi: 10.3390/metabo10090373 32948079 PMC7570375

[B20] Lee H. Kang W. Ha Y. Jung Y. Bin Y. Park T. (2025). Phenylacetaldehyde attenuates Cutibacterium acnes-induced inflammation in keratinocytes and monocytes. Int. Immunopharmacol. 158, 114885. doi: 10.1016/j.intimp.2025.114885 40383096

[B21] Legrand M. Clark A. T. Neyra J. A. Ostermann M. (2024). Acute kidney injury in patients with burns. Nat. Rev. Nephrol. 20, 188–200. doi: 10.1038/s41581-023-00769-y 37758939 PMC13340345

[B22] Lelou E. Corlu A. Nesseler N. Rauch C. Mallédant Y. Seguin P. . (2022). The role of catecholamines in pathophysiological liver processes. Cells 11. doi: 10.3390/cells11061021 35326472 PMC8947265

[B23] Liu X. Wu Y. Liu Y. Qian W. Huang L. Wu Y. . (2025). UPLC-MS/MS-based serum metabolomics analysis for comprehensive pathological myopia profiling. Exp. Eye Res. 251, 110152. doi: 10.1016/j.exer.2024.110152 39603320

[B24] Loss M. Wedler V. Künzi W. Meuli-Simmen C. Meyer V. E. (2000). Artificial skin, split-thickness autograft and cultured autologous keratinocytes combined to treat a severe burn injury of 93% of TBSA. Burns 26, 644–652. doi: 10.1016/s0305-4179(00)00045-0 10925189

[B25] Malachowska B. Yang W. L. Qualman A. Muro I. Boe D. M. Lampe J. N. . (2023). Transcriptomics, metabolomics, and in-silico drug predictions for liver damage in young and aged burn victims. Commun. Biol. 6, 597. doi: 10.1038/s42003-023-04964-2 37268765 PMC10238406

[B26] Mercola J. D'Adamo C. R. (2023). Linoleic acid: a narrative review of the effects of increased intake in the standard American diet and associations with chronic disease. Nutrients 15. doi: 10.3390/nu15143129 37513547 PMC10386285

[B27] Molfino A. Imbimbo G. Gallicchio C. Muscaritoli M. (2024). Tryptophan metabolism and kynurenine metabolites in cancer: systemic nutritional and metabolic implications. Curr. Opin. Clin. Nutr. Metab. Care 27, 316–321. doi: 10.1097/mco.0000000000001021 38386476

[B28] Moreira E. Burghi G. Manzanares W. (2018). Update on metabolism and nutrition therapy in critically ill burn patients. Med. Intensiva (Engl Ed) 42, 306–316. doi: 10.1016/j.medine.2018.04.004 28951113

[B29] Nakazawa H. Wong L. P. Shelton L. Sadreyev R. Kaneki M. (2022). Farnesysltransferase inhibitor prevents burn injury-induced metabolome changes in muscle. Metabolites 12. doi: 10.3390/metabo12090800 36144205 PMC9506277

[B30] Parihar A. Parihar M. S. Milner S. Bhat S. (2008). Oxidative stress and anti-oxidative mobilization in burn injury. Burns 34, 6–17. doi: 10.1016/j.burns.2007.04.009 17905515

[B31] Pashkevich N. I. Vilyanen D. V. Marcinkevich A. F. Borisova-Mubarakshina M. M. Osochuk S. S. (2024). The effect of liposomes of various compositions on the skin and its derivatives after II-IIIA degree thermal burns. Acta Naturae 16, 67–76. doi: 10.32607/actanaturae.27329 38698959 PMC11062103

[B32] Ploder M. Spittler A. Kurz K. Neurauter G. Pelinka L. E. Roth E. . (2010). Accelerated tryptophan degradation predicts poor survival in trauma and sepsis patients. Int. J. Tryptophan Res. 3, 61–67. doi: 10.4137/ijtr.s3983 22084588 PMC3195245

[B33] Rehou S. de Brito Monteiro L. Auger C. Knuth C. M. Abdullahi A. Stanojcic M. . (2023). Propranolol normalizes metabolomic signatures thereby improving outcomes after burn. Ann. Surg. 278, 519–529. doi: 10.1097/sla.0000000000005973 37389480

[B34] Ryan M. J. Raby E. Whiley L. Masuda R. Lodge S. Nitschke P. . (2024). Nonsevere burn induces a prolonged systemic metabolic phenotype indicative of a persistent inflammatory response postinjury. J. Proteome Res. 23, 2893–2907. doi: 10.1021/acs.jproteome.3c00516 38104259 PMC11302432

[B35] She H. Du Y. Du Y. Tan L. Yang S. Luo X. . (2023). Metabolomics and machine learning approaches for diagnostic and prognostic biomarkers screening in sepsis. BMC Anesthesiol. 23, 367. doi: 10.1186/s12871-023-02317-4 37946144 PMC10634148

[B36] Sommerhalder C. Blears E. Murton A. J. Porter C. Finnerty C. Herndon D. N. (2020). Current problems in burn hypermetabolism. Curr. Probl. Surg. 57, 100709. doi: 10.1016/j.cpsurg.2019.100709 32033707 PMC7822219

[B37] Song J. de Libero J. Wolf S. E. (2014). Hepatic autophagy after severe burn in response to endoplasmic reticulum stress. J. Surg. Res. 187, 128–133. doi: 10.1016/j.jss.2013.09.042 24209807 PMC4169053

[B38] Stanojcic M. Abdullahi A. Rehou S. Parousis A. Jeschke M. G. (2018). Pathophysiological response to burn injury in adults. Ann. Surg. 267, 576–584. doi: 10.1097/sla.0000000000002097 29408836 PMC8966302

[B39] Strasser B. Sperner-Unterweger B. Fuchs D. Gostner J. M. (2017). Mechanisms of inflammation-associated depression: immune influences on tryptophan and phenylalanine metabolisms. Curr. Top. Behav. Neurosci. 31, 95–115. doi: 10.1007/7854_2016_23 27278641

[B40] Strauss J. C. Haskey N. Ramay H. R. Ghosh T. S. Taylor L. M. Yousuf M. . (2023). Weighted gene co-expression network analysis identifies a functional guild and metabolite cluster mediating the relationship between mucosal inflammation and adherence to the Mediterranean diet in ulcerative colitis. Int. J. Mol. Sci. 24. doi: 10.3390/ijms24087323 37108484 PMC10138710

[B41] Su S. Zhang Y. Wu D. Wang C. Hu J. Wei Y. . (2024). (1)H-nuclear magnetic resonance analysis reveals dynamic changes in the metabolic profile of patients with severe burns. Burns Trauma 12, tkae007. doi: 10.1093/burnst/tkae007 38756185 PMC11097601

[B42] Sun J. K. Sun F. Wang X. Yuan S. T. Zheng S. Y. Mu X. W. (2015). Risk factors and prognosis of hypoalbuminemia in surgical septic patients. PeerJ 3, e1267. doi: 10.7717/peerj.1267 26557421 PMC4636415

[B43] TeSlaa T. Ralser M. Fan J. Rabinowitz J. D. (2023). The pentose phosphate pathway in health and disease. Nat. Metab. 5, 1275–1289. doi: 10.1038/s42255-023-00863-2 37612403 PMC11251397

[B44] Tsuji A. Ikeda Y. Yoshikawa S. Taniguchi K. Sawamura H. Morikawa S. . (2023). The tryptophan and kynurenine pathway involved in the development of immune-related diseases. Int. J. Mol. Sci. 24. doi: 10.3390/ijms24065742 36982811 PMC10051340

[B45] Wang Y. Luo Y. Yang S. Jiang M. Chu Y. (2023). LC-MS/MS-based serum metabolomics and transcriptome analyses for the mechanism of augmented renal clearance. Int. J. Mol. Sci. 24. doi: 10.3390/ijms241310459 37445637 PMC10341629

[B46] Wei B. Zheng J. Chai J. Huang J. Duan H. Han S. . (2025). Metabolomic and proteomic profiling of a burn-hemorrhagic shock swine model reveals a metabolomic signature associated with fatal outcomes. Eur. J. Med. Res. 30, 10. doi: 10.1186/s40001-024-02245-0 39773520 PMC11706163

[B47] Willis M. L. Mahung C. Wallet S. M. Barnett A. Cairns B. A. Coleman L. G. . (2022). Plasma extracellular vesicles released after severe burn injury modulate macrophage phenotype and function. J. Leukoc. Biol. 111, 33–49. doi: 10.1002/jlb.3mia0321-150rr 34342045 PMC8716518

[B48] Wischmeyer P. E. (2008). Glutamine: role in critical illness and ongoing clinical trials. Curr. Opin. Gastroenterol. 24, 190–197. doi: 10.1097/mog.0b013e3282f4db94 18301270

[B49] Wischmeyer P. E. (2019). Glutamine in burn injury. Nutr. Clin. Pract. 34, 681–687. doi: 10.1002/ncp.10362 31270877

[B50] Yang Y. Chen Q. Fan S. Lu Y. Huang Q. Liu X. . (2024). Glutamine sustains energy metabolism and alleviates liver injury in burn sepsis by promoting the assembly of mitochondrial HSP60-HSP10 complex via SIRT4 dependent protein deacetylation. Redox Rep. 29, 2312320. doi: 10.1080/13510002.2024.2312320 38329114 PMC10854458

[B51] Yang Y. Su S. Zhang Y. Wu D. Wang C. Wei Y. . (2022). Effects of different ratios of carbohydrate-fat in enteral nutrition on metabolic pattern and organ damage in burned rats. Nutrients 14. doi: 10.3390/nu14173653 36079913 PMC9460118

[B52] Zhu Y. Chen X. Lu Y. Xia L. Fan S. Huang Q. . (2022). Glutamine mitigates murine burn sepsis by supporting macrophage M2 polarization through repressing the SIRT5-mediated desuccinylation of pyruvate dehydrogenase. Burns Trauma 10, tkac041. doi: 10.1093/burnst/tkac041 36601059 PMC9801296

