# Supplementary figures and images for: Integrated serum metabolomics reveals severity-associated exploratory metabolic signatures in burn patients
Source: Front Physiol. 2026 Jun 17;17:1846440. doi: 10.3389/fphys.2026.1846440 (PMC13318791; doi:10.3389/fphys.2026.1846440)

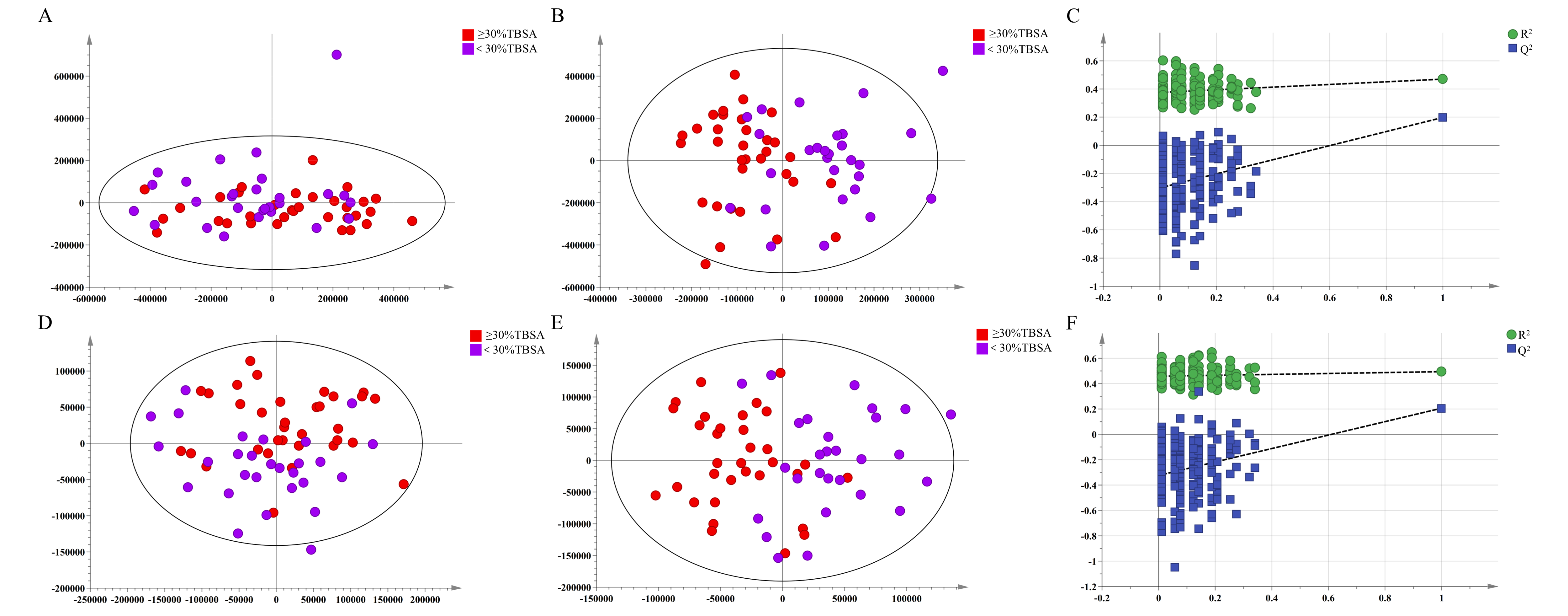

Supplement: Supplementary Figure 1 — Direct multivariate comparison between burn patients with ≥30% TBSA (n=33) and <30% TBSA (n=28). PCA and OPLS-DA analyses were performed to evaluate severity-associated metabolic differences between the two burn groups under both ESI+ and ESI− modes. PCA was used for unsupervised clustering analysis, whereas OPLS-DA was applied for supervised discrimination analysis. Each point represents an individual serum sample. Permutation testing was performed to assess model stability and potential overfitting. (A) PCA scores (ESI+); (B) OPLS-DA scores (ESI+); (C) Permutation test (ESI+); (D) PCA scores (ESI−); (E) OPLS-DA scores (ESI−); (F) Permutation test (ESI−). [file Image1.tif]

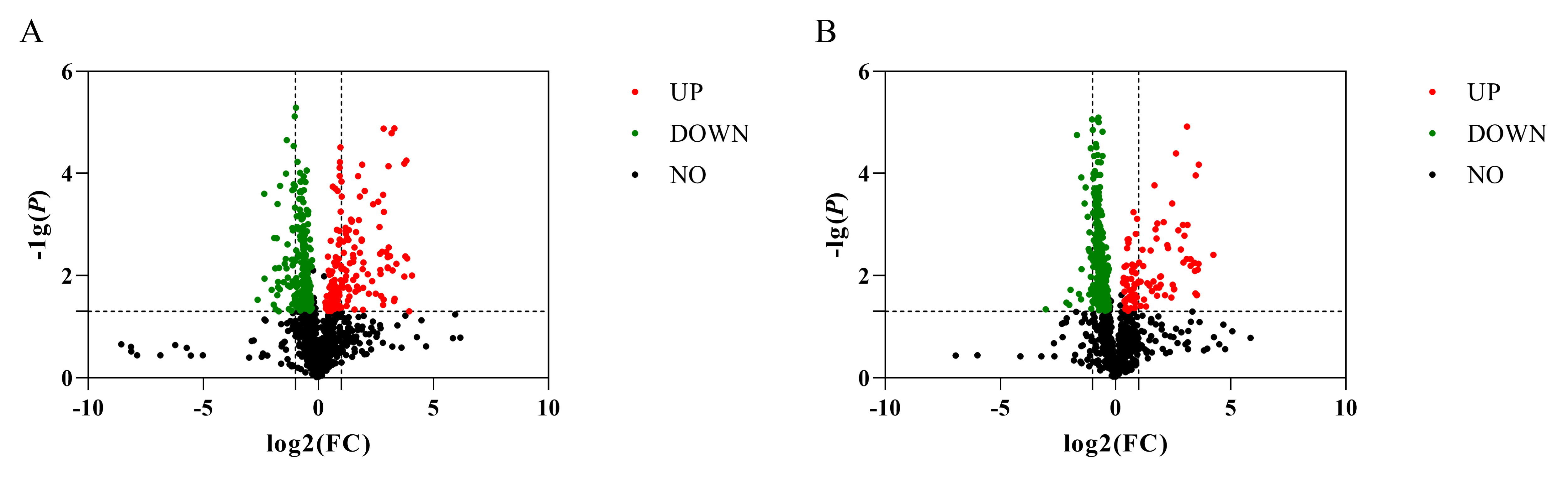

Supplement: Supplementary Figure 2 — Volcano plot of differential metabolites between TBSA ≥30% and TBSA <30% burn groups. Differential metabolites were screened based on VIP >1, P < 0.05, and FC >1.2 or <0.83. Red dots indicate metabolites with significantly increased abundance, green dots indicate metabolites with significantly decreased abundance, and black dots represent non-significant metabolites. (A) ESI+; (B) ESI−. [file Image2.tif]
